# The Russian version of the Abbreviated Math Anxiety Scale: psychometric properties in adolescents aged 13–16 years

**DOI:** 10.3389/fpsyg.2023.1275212

**Published:** 2023-12-15

**Authors:** Julia Marakshina, Anna Pavlova, Victoria Ismatullina, Timofey Adamovich, Sofia Mironets, Maria A. Sitnikova, Marina Lobaskova, Sergey Malykh

**Affiliations:** ^1^Center for Interdisciplinary Research in the Educational Sciences, Russian Academy of Education, Moscow, Russia; ^2^Developmental Behavioral Genetics Laboratory, Federal Research Centre of Psychological and Interdisciplinary Studies, Moscow, Russia

**Keywords:** math anxiety, AMAS, factor validity, internal consistency, adolescents

## Abstract

This study is the first to assess the internal consistency and factor validity of the Abbreviated Math Anxiety Scale (AMAS) in a sample of Russian adolescents as well as gender differences and gender invariance. The study included 4,218 adolescents in grades 7–9 (*M* = 14.23, SD = 0.92). Internal consistency, measured with Cronbach’s alpha, was high. Analysis of the factor structure revealed the best correspondence of the second-order factor model, which included two scales (learning math anxiety and math evaluation anxiety) and the general scale of math anxiety. There were greater gender differences in the all three scales. Analysis of gender invariance demonstrated that the mathematics anxiety construct was uniform in boys and girls. These findings confirm the reliable psychometric properties and validity of the AMAS, enabling its use in adolescents.

## 1 Introduction

Mathematics anxiety (MA) can be defined as a feeling of tension, apprehension, or fear that interferes with math performance. It manifests as negative emotions when completing operations with numbers and other mathematical material (Richardson and Suinn, 1972). People with mathematics anxiety have difficulties solving arithmetic and other mathematical problems and memorizing numbers because during these activities, they experience fear or anxiety. This state can occur both in mathematics lessons and in everyday life (for example, when counting change). MA demonstrates negative associations with mathematical achievement (Dowker et al., 2016; Zhang et al., 2019). The direction of the relationship between MA and math performance has been debated, leading to several explanations (Meece et al., 1990; Ma and Xu, 2004; Ashcraft and Krause, 2007). According to the Cognitive Impairment Model (Maloney et al., 2010, 2011, 2012; Ferguson et al., 2015), or “Deficit Theories” (Carey et al., 2016), initially low level of numerical and spatial abilities or mathematical achievement leads to development of MA. However, one study does not support this hypothesis (Field et al., 2019). Another possible explanation is the debilitating anxiety model (Carey et al., 2016). This is supported by evidence that measures to reduce MA led to increased scores on subsequent math tests (Faust, 1996; Schmader et al., 2008; Park et al., 2014). Finally, the “Reciprocal Effect” hypothesis was proposed (Carey et al., 2016). According to this hypothesis, the development of basic abilities such as reasoning, number understanding and poor academic performance cause the MA, and MA may contribute to decreased academic performance. Reciprocal relationships between MA and mathematical achievement have been found in various studies (Pekrun, 2006; Ashcraft and Krause, 2007; Luo et al., 2014; Gunderson et al., 2018; Schillinger et al., 2018).

Research shows that math anxiety often correlates with general anxiety (Hembree, 1990). Moderate correlations are found by other researchers (Ma, 1999; Wang et al., 2014; Malanchini et al., 2017). The contribution of genetic differences in general anxiety to genetic differences in MA has been reported (Wang et al., 2014). Simply put, an anxious person also worries about math. However, these are different phenomena; people who are not inclined to be nervous about other things can show mathematics anxiety. In support of this fact, it has been reported that different measures of MA are more strongly correlated with each other, rather than the constructs of general and math anxiety (Ashcraft and Ridley, 2005).

In addition, some researchers believe that it is necessary to discuss subject anxiety in general or science anxiety (Mallow, 2006; Megreya et al., 2021). That is, the anxiety that manifests in different people related to different disciplines (e.g., languages or physics). However, this is only one theory. Alternatively, MA may be primarily associated with the testing context, referred to as test anxiety (Fournier et al., 2017). These states can be distinguished.

The prevalence of MA varies in different studies. Some authors have reported that one in ten individuals suffers from MA, while others have reported that almost 70% of people experience such difficulties (Richardson and Suinn, 1972; Betz, 1978; Ashcraft and Moore, 2009; Johnston-Wilder et al., 2014). Difference in estimates is related to how to divide the population for assessment (for example, which age groups), how strict an assessment criterion to use. This is due to the fact that MA is a continuous measure and agreement is required on which point should be considered the starting point for MA. In addition, differences in prevalence may be due to differences in the constructs that are measured, such as trait and state math anxiety (Orbach et al., 2020).

An important aspect of MA is the age of onset. The authors report the occurrence of math anxiety in children at the age corresponding to attending kindergarten (Lu et al., 2021). Math anxiety starts even before elementary school, but most studies have shown that it increases in severity by high school (Dowker et al., 2016). Psychophysiological studies have shown that as early as the age of 7–9 years, while learning mathematics, brain areas associated with the occurrence of MA can be activated in children (Young et al., 2012). However, gender differences in the level of MA appear later. Most studies suggest that there are no gender differences in MA in primary school children (Dowker et al., 2012; Wu et al., 2012; Harari et al., 2013). It is argued that gender differences in math anxiety appear only in adolescents. Some researchers associate differences in MA with gender stereotypes that arise in the family or society. Gender stereotypes claim that women are worse at math than men (Hembree, 1990; Beilock et al., 2007; Goetz et al., 2013). At the same time, it is important to note that men and women in countries where education is equally available to both genders demonstrate few differences in mathematics achievement (Spelke, 2005). The relationship between MA and academic achievement is mediated by different variables for males and females. In males, MA can be caused by test anxiety and basic math scores (Miller and Bichsel, 2004; Devine et al., 2012). The contribution of visuospatial memory to the structure of these relationships is tested, since the influence of MA to visuospatial memory is possible (Ganley and Vasilyeva, 2014). In addition, math anxiety may increase on average in a population with age. A possible mechanism may be the complexity of mathematical material in the classroom or the growing role of gender stereotypes and their influence on problem solving (in girls). In other words, girls, influenced by gender stereotypes, may rate their math abilities lower and may be more worried about math. Additionally, distinguishing among adolescents according to MA severity is important. In addition, math anxiety may increase with age, like other types of anxiety, which may cause an increase in anxiety disorders in adolescents (Kiessler et al., 2005). Overall, there are high demands on the measuring instrument used in adolescents.

To date, several questionnaires have been developed to identify MA. The first Mathematics Anxiety Rating Scale (MARS) was developed by Frank Richardson and Richard Suinn in 1972 as “a measure of anxiety associated with the single area of the manipulation of numbers and the use of mathematical concepts” (Richardson and Suinn, 1972, p.551). The scale consisted of 98 questions. Several variants of the MARS have been developed, including the revised MARS (MARS-R) (Plake and Parker, 1982). The MARS-R is a version of the MARS (Richardson and Suinn, 1972) consisting of 24 items about situations related to mathematics; subjects rate each item on a 5-point scale to indicate the degree of excitement, fear and nervousness. The Abbreviated Math Anxiety Scale (AMAS) was developed from the MARS-R (Hopko et al., 2003). The Abbreviated Math Anxiety Scale (AMAS) is a 9-item self-report questionnaire that measures math anxiety. The AMAS has excellent internal reliability (0.90) (Hopko et al., 2003). Each item consists of a description of an event, such as “Watching the teacher work on an algebraic equation on the blackboard,” and participants rate the anxiety induced by this event on a scale from 1 (low) to 5 (high). A high score on this questionnaire may indicate the presence of MA. The scale is intended for use in both adults and adolescents aged 11–16. The factor structure of the scale differs among different populations. The two-factor structure has predominantly demonstrated consistency. Initially, the AMAS had a two-factor structure: learning math anxiety (LMA) and math evaluation anxiety (MEA). However, a bifactorial structure has also been described, including the two factors and a common factor that encompasses them (Cohen and Limbers, 2022). Additionally, in some studies, structures involving two factors, where one of the questions applies to both, have shown high factor validity (Cipora et al., 2015, 2017; Schillinger et al., 2018; Martín-Puga et al., 2022). The AMAS was developed for school children and university students in different countries: the USA, Italy, Spain, Germany, Poland, and Serbia (Hopko et al., 2003; Cipora et al., 2015, 2017; Schillinger et al., 2018; Primi et al., 2020; Milovanović and Branovački, 2021; Cohen and Limbers, 2022; Martín-Puga et al., 2022). For an overview of known AMAS adaptations, see recent study of Martín-Puga and colleagues (Martín-Puga et al., 2022). However, the use of the AMAS has not been evaluated in Russia. Moreover, its use in adolescents to diagnose MA remains unclear. This requires an assessment of the psychometric properties of the AMAS in a sample of Russian adolescents. This can allow us to identify the presence of academic stress associated with MA in adolescents. Thus, the aims of this study were to (1) evaluate the factorial validity of the AMAS, (2) assess the reliability of the scale in a sample of Russian adolescents, (3) estimate the external validity of the AMAS, and (4) determine measurement invariance and gender differences.

## 2 Method

### 2.1 Sample

Initially, 4,218 participants completed the questionnaire. We excluded data from participants younger than 12 and those with missing data on their gender. Thus, the final sample consisted of 4,088 adolescents. The age ranged from 12 to 16 years (mean = 14.23; median = 14.0, standard deviation = 0.92). There were 1906 (47%) males and 2,182 (53%) females. The grades ranged from 7 to 9. A total of 655 adolescents were in 7th grade, 1,752 were in 8th grade, and 1,681 were in 9th grade.

### 2.2 Instruments

The Abbreviated Math Anxiety Scale, AMAS (9-item). The AMAS was adapted from Hopko et al. (2003). Translation, back translation and adaptation of the scale were carried out by the Laboratory of Cognitive and Interdisciplinary Research, Sirius Educational Center. It consists of 9 items, with 5 items on the learning math anxiety subscale and 4 items on the math evaluation anxiety subscale. Respondents are instructed to rate each statement in terms of how much anxiety they feel in each of the situations described. Answers are provided on a Likert scale from 1 (low anxiety) to 5 (high anxiety).

State–Trait Anxiety Inventory: trait anxiety subscale, STAI-T (Spielberger et al., 1970). This subscale is one of the subscales of the State–Trait Anxiety Inventory (STAI) developed by Spilberger. The STAI-T assesses relatively stable aspects of anxiety propensity, including general states of calmness, confidence, and security. Participants report how they usually feel. Ratings range from “almost never” to “almost always.” Translation, back translation and adaptation of the scale were carried out by the Laboratory of Cognitive and Interdisciplinary Research, Sirius Educational Center The Cronbach’s alpha coefficient for STAI-T subscale was 0.765.

Perceived Difficulty of Math (subscale of a questionnaire used in “Gender stereotypes and incremental beliefs about STEM”) (Ismatullina et al., 2022). This subscale is used to identify difficulties associated with the study of mathematics reported by adolescents. This scale includes 4 items (“I usually do well in math” (reverse-coded), “Math is harder for me than for many of my classmates,” “Studying math gives me anxiety,” and “Math is harder for me than other subjects”). Participants rate all items on the same 4-point Likert scale (with 2 negative and 2 positive ratings). Cronbach’s alpha (0.8) of the scale indicated good internal consistency.

### 2.3 Procedure

The data were collected during online testing conducted in the classroom at school. Parental consent for adolescents to participate in testing was obtained. The study was approved by the Ethics Committee of the Psychological Institute of the Russian Academy of Education.

### 2.4 Statistical analysis

Data analysis was performed using R 4.2.1 version (descriptive statistics, confirmatory factor analysis, evaluation of internal consistency, evaluation of gender differences, correlation analysis) and JASP (structural equation modeling for measurement invariance). Confirmatory factor analysis (CFA) was used to examine the factor structure of the AMAS. The fit of the model to the empirical data was tested using a number of criteria. Good model fit was defined by a standardized root mean square (SRMR) of <0.08, Tucker–Lewis index (TLI) values close to 1, comparative fit index (CFI) >0.95, and root mean square error of approximation (RMSEA) <0.08 (Hu and Bentler, 1999; Byrne, 2001; Kline, 2005; Brown, 2006). For the CFA, the WLSMV estimator was used. Cronbach’s alpha was used to assess internal consistency. Alpha values above 0.7 were used to indicate good internal consistency (Nunnally, 1978). Spearman’s correlation analysis was applied. An independent-sample t-test was performed to evaluate differences between the genders. The configural and metric invariance using the “auto” estimator in the SEM function in JASP was evaluated. Fit indices such as ΔCFI and ΔRMSEA were assessed. Configural invariance was assessed by CFI and RMSEA. ΔCFI <0.010 and ΔRMSEA <0.015 indicated metric invariance (Cheung and Rensvold, 2002; Chen, 2007).

## 3 Results

### 3.1 Descriptive statistics

During the analysis, descriptive statistics were calculated. The results are presented in Table 1. LMA was less pronounced than MEA. Overall, the AMAS scores exhibited positive skew. Pearson’s correlation coefficient of the relationship between LMA and MEA scores was 0.50.

**Table 1:** Descriptive statistics for LMA, MEA, and AMAS total scores.

	Number of items	Mean	Std	Median	Max
LMA	5	3.49	4.40	2	20
MEA	4	7.39	4.23	8	16
AMAS total	9	10.88	7.47	10	36

### 3.2 Confirmatory factor analysis and internal consistency

Four models were verified: one-factor, two-factor, bifactor (with two factors and a general factor) and second-order factor model in which two specific factors were correlated with the general factor. The following goodness-of-fit indices of CFA models were observed: for one-factor model *χ*2 (113465.405) = 4129.798, *p* < 0.001, CFI = 0.964, TLI = 0.952, SRMR = 0.150, RMSEA = 0.193, 90% CI [0.188; 0.198]; for two-factor model *χ*2 (113465.405) = 1232.147, *p* < 0.001, CFI = 0.989, TLI = 0.985, SRMR = 0.078, RMSEA = 0.107, 90% CI [0.102; 0.112]; for bifactor model *χ*2 (113465.405) = 166.964, *p* < 0.001, CFI = 0.999, TLI = 0.997, SRMR = 0.029, RMSEA = 0.045, 90% CI [0.039; 0.051]; for second-order factor model *χ*2 (113465.405) = 110.848, *p* < 0.001, CFI = 0.999, TLI = 0.998, SRMR = 0.023, RMSEA = 0.040, 90% CI [0.033; 0.047]. As can be seen, the second-order factor model provided the best fit. Factor loadings of AMAS items are presented in Table 2.

**Table 2:** Standardized factor loadings of the second-order factor model.

Title 1	Learning math anxiety	Math evaluation anxiety	General math anxiety
1. Having to use the tables in the back of a math book. Используя таблицы в конце учебника по математике.	0.111		0.774
3. Watching a teacher work an algebraic equation on the blackboard. Наблюдая, как преподаватель объясняет алгебраическое уравнение на доске.	0.202		0.876
6. Listening to a lecture in math class. Слушая лекцию на занятии по математике.	0.023		0.924
7. Listening to another student explain a math formula. Слушая, как другой студент объясняет математическую формулу.	0.006		0.890
9. Starting a new chapter in a math book. Начиная новую главу по математике.	0.240		0.857
2. Thinking about an upcoming math test 1 day before. Думая накануне о предстоящем тесте по математике.		0.661	0.610
4. Taking an examination in a math course. Выполняя экзамен по математике.		0.854	0.372
8. Being given a “pop” quiz in math class. Выполняя внеплановую контрольную на занятии по математике.		0.738	0.479
5. Being given a homework assignment of many difficult problems that is due the next class meeting. Получая домашнюю работу с большим количеством трудных задач, которую нужно решить к следующему занятию.		0.433	0.653

Cronbach’s alpha was evaluated to assess internal consistency. Cronbach’s alpha was 0.89 for LMA, 0.84 for MEA, and 0.88 for the general scale.

### 3.3 Construct validity

Spearman’s correlation analyses were conducted for the relationships of the AMAS Total score with STAI-T scores and Perceived Difficulty of Math in the sample of 2,434 participants. Significant correlations were observed between AMAS Total and STAI-T scores (*r* = 0.38, *p* < 0.001), between LMA and STAI-T scores (*r* = 0.34, *p* < 0.001), between MEA and STAI-T scores (*r* = 0.32, *p* < 0.001), between AMAS Total and Perceived Difficulty of Math (*r* = 0.27, *p* < 0.001), between LMA and Perceived Difficulty of Math (*r* = 0.24, *p* < 0.001), between MEA and Perceived Difficulty of Math (*r* = 0.22, *p* < 0.001).

### 3.4 Gender differences and measurement invariance across genders

First, gender differences were analyzed. The results are presented in Table 3. Overall, girls reported higher math anxiety, which is consistent with previous studies. Second, measurement invariance was analyzed. Measurement invariance across genders was evaluated to assess the applicability of the AMAS in both male and female groups. Initially, the model parameters were estimated for each gender, i.e., configural invariance was tested. This model may be useful to understand whether our second-order factor model is applicable to the comparison of groups. Next, metric invariance was tested. It means that the factor loadings between groups are the same, i.e., the relationship between latent variable scores and items is similar between compared groups. This test answers the question of whether the difference in model fit between the two models is significant. In other words, the null hypothesis that the factor loadings are equal for both genders is tested. Model 1 (Table 4) demonstrates configural invariance with a good fit to the data (CFI = 0.999, RMSEA = 0.042). Model 2 was used to evaluate metric invariance and demonstrated a good fit to the data (ΔCFI = 0.001, ΔRMSEA = 0.004). However, *p* < 0.001 indicates that the null hypothesis should be rejected. Thus, our data demonstrate gender invariance in the Model 1 (configural invariance).

**Table 3:** Gender differences and descriptive statistics for LMA, MEA, and AMAS total scores.

	Male		Female		Mean difference	*T*-test statistics	*p*-value
	M	Sd	M	Sd			
LMA	2.61	3.18	2.90	3.78	0.29	−2.16	0.031
MEA	6.48	4.12	8.40	4.08	1.92	−13.43	<0.001
AMAS total	9.09	7.03	11.30	6.84	2.21	−9.15	<0.001

**Table 4:** Model fit indices for measurement invariance across gender groups.

	CFI	RMSEA	Baseline test			Difference test				
			*χ* ^2^	Df	*p*	ΔCFI	ΔRMSEA	Δ*χ*^2^	Δdf	*p*
Model 1	0.999	0.042	164.621	36	< 0.001					
Model 2	0.998	0.046	267.615	51	< 0.001	0.001	0.004	93.702	15	< 0.001

## 4 Discussion

In this study, the psychometric properties of the AMAS were tested in a large sample of Russian adolescents in grades 7–9. This is the first large-scale study on AMAS psychometric properties conducted in Russia.

First, we examined the factor validity of the AMAS. We tested four models previously described in studies of this scale: one-factor, two-factor, bifactor, and second-order factor models. The second-order factor model showed the best fit to the empirical data. Thus, the structure of the questionnaire is described by two scales: LMA and MEA. In this model the scales correlated with the general scale of mathematics anxiety. This factor structure has been described by a number of other studies in both children and adults (e.g., Sadiković et al., 2018; Cohen and Limbers, 2022). Given that the model not only demonstrated a good empirical fit to the data, but also was consistent with theoretical concepts of a two-factor structure, and that the correlated factor model was supported by other studies (Martín-Puga et al., 2022), it was selected as the best suitable. Thus, the results obtained from a sample of Russian adolescents are consistent with the results of studies conducted in other countries. In this case, items scores were distributed in the expected manner. The latent construct of learning anxiety, as measured by the LMA subscale, included Items 1, 3, 6, 7, and 9. The latent construct of test anxiety, described by the MEA subscale, consisted of Items 2, 4, 5, and 8.

The internal consistency of the AMAS was high, as assessed using Cronbach’s alpha coefficient. This result suggests that the AMAS can be applied in adolescents aged 13–16 years.

The construct validity of the AMAS was assessed using two scales. One of them was the STAI-T, a scale that is classically used in research to assess trait anxiety. Previous study of Cipora and colleagues has shown that trait and math anxiety are correlated (Cipora et al., 2015). This is consistent with our findings that the AMAS and STAI-T scores were correlated at *r* = 0.32–0.38. The correlation was weak but significant. Similar correlation magnitudes have been obtained in the study by Cipora and colleagues (Cipora et al., 2015). Another scale, Perceived Difficulty of Math, also showed weak (*r* = 0.22–0.27) but significant correlations with the AMAS scores. A possible explanation of weak correlation may be that the Perceived Difficulty of Math Scale includes not only the anxiety component but also other constructs (in particular, self-esteem). However, the correlations were expectedly positive and show a direct relationship between the constructs. In general, the obtained results support the construct validity of the AMAS on the basis of significant positive correlations between AMAS and STAI-T, AMAS and Perceived Difficulty of Math Scale.

An assessment of gender differences showed that girls had higher scores on all three scales. Significant differences were obtained on the all three scales of mathematics anxiety. Girls tended to be more anxious than boys in testing contexts and, in general, had more severe mathematics anxiety. This is consistent with previous studies on this topic (Szczygiel, 2020). On the other hand, girls demonstrated higher anxiety in learning situations, for example, at the lessons. In general, women and girls report higher anxiety (Xie et al., 2019). This also applies to trait anxiety and specific types of anxiety, such as object and test anxiety (Hembree, 1990; Ashcraft et al., 1998). Mathematics anxiety is also more pronounced in women. Moreover, extreme manifestations of anxiety associated with the development of anxiety disorders are also observed more often in women. Such differences are explained, on the one hand, by biological reasons (in particular, gender differences in the brain) (McLean and Anderson, 2009). On the other hand, social causes may also play a role. For example, the development of anxiety can be influenced by gender stereotypes (Tomasetto, 2019). Thus, stereotypes may be a possible explanation of negative perceptions in girls about their success in mathematics. In turn, these representations can trigger anxiety when solving mathematical problems. Math anxiety can predict future math achievement in females in comparison with males (Casanova et al., 2021). However, the construct of mathematics anxiety itself did not differ between boys and girls. This conclusion is supported by the demonstrated gender invariance. Our results indicate configural invariance across genders. This means that the model describing AMAS is suitable for both genders.

А possible limitation of this study may be the lack of assessments of test–retest reliability. Future research could develop norms for this scale. Another limitation is related to the deficit of measurements in Russian that may be used for external validation. Thus, the further direction of research may include development of other instruments for MA estimation. Among possible measurements for this aim state MA questionnaire (Orbach et al., 2020), express one-item scale (Núñez-Peña et al., 2014) or physiological assessment (Haase et al., 2019) may be designed.

To sum up, the psychometric properties of the AMAS suggest that it can be used to measure mathematics anxiety in Russian adolescents aged 13–16 years. This was confirmed by the high internal consistency and factor validity. In the present study, gender differences were observed, which are similar to those previously reported. Gender invariance was demonstrated, suggesting that the construct of mathematics anxiety in boys and girls is similar. In general, the results obtained are consistent with reports on AMAS scores in other countries. This expands our knowledge of the construct of mathematics anxiety in populations around the world and suggests opportunities to use the AMAS in cross-cultural studies. In general, these findings contribute to the improvement of mathematical education.

## Data availability statement

The raw data supporting the conclusions of this article will be made available by the authors, without undue reservation.

## Ethics statement

The studies involving humans were approved by the Ethics Committee of the Psychological Institute of the Russian Academy of Education (protocol code 2020/4-1, date of approval 02 April 2020). The studies were conducted in accordance with the local legislation and institutional requirements. Written informed consent for participation in this study was provided by the participants’ legal guardians/next of kin.

## Author contributions

JM: Conceptualization, Writing – original draft. AP: Writing – original draft, Formal analysis. VI: Investigation, Writing – review & editing. TA: Software, Writing – review & editing. SMi: Investigation, Writing – review & editing. MS: Investigation, Writing – review & editing. ML: Investigation, Writing – review & editing. SMa: Writing – review & editing, Supervision.

## References

[ref1] Ashcraft M. H. Kirk E. P. Hopko D. (1998). “On the cognitive consequences of mathematics anxiety” in The development of mathematical skills. ed. Donlan C. (United Kingdom: Psychology Press/Taylor & Francis), 175–196.

[ref2] Ashcraft M. H. Krause J. A. (2007). Working memory, math performance, and math anxiety. Psychon. Bull. Rev. 14, 243–248. doi: 10.3758/BF03194059 17694908

[ref3] Ashcraft M. H. Moore A. M. (2009). Mathematics anxiety and the affective drop in performance. J. Psychoeduc. Assess. 27, 197–205. doi: 10.1177/0734282908330580

[ref4] Ashcraft M. H. Ridley K. S. (2005). “Math anxiety and its cognitive consequences: a tutorial review” in The handbook of mathematical cognition. ed. Campbell J. I. D. (New York, NY: Psychology Press), 315–327.

[ref5] Beilock S. L. Rydell R. J. McConnell A. R. (2007). Stereotype threat and working memory: mechanisms, alleviation, and spillover. J. Exp. Psychol. Gen. 136, 256–276. doi: 10.1037/0096-3445.136.2.256, PMID: 17500650

[ref6] Betz N. E. (1978). Prevalence, distribution, and correlates of math anxiety in college students. J. Couns. Psychol. 25, 441–448. doi: 10.1037/0022-0167.25.5.441

[ref7] Brown T. A. (2006). Confirmatory factor analysis for applied research (New York, New York).

[ref8] Byrne B. M. (2001). Structural equation modeling with AMOS, EQS, and LISREL: comparative approaches to testing for the factorial validity of a measuring instrument. Int. J. Test. 1, 55–86. doi: 10.1207/S15327574IJT0101_4

[ref10] Carey E. Hill F. Devine A. Szücs D. (2016). The chicken or the egg? The direction of the relationship between mathematics anxiety and mathematics performance. Front. Psychol. 6:1987. doi: 10.3389/fpsyg.2015.01987, PMID: 26779093 PMC4703847

[ref11] Casanova S. Vukovic R. K. Kieffer M. J. (2021). Do girls pay an unequal price? Black and Latina girls' math attitudes, math anxiety, and mathematics achievement. J. Appl. Dev. Psychol. 73:101256. doi: 10.1016/j.appdev.2021.101256

[ref12] Chen F. F. (2007). Sensitivity of goodness of fit indexes to lack of measurement invariance. Struct. Equ. Model. Multidiscip. J. 14, 464–504. doi: 10.1080/10705510701301834

[ref13] Cheung G. W. Rensvold R. B. (2002). Evaluating goodness-of-fit indexes for testing measurement invariance. Struct. Equ. Model. 9, 233–255. doi: 10.1207/S15328007SEM0902_5

[ref14] Cipora K. Szczygieł M. Willmes K. Nuerk H. C. (2015). Math anxiety assessment with the abbreviated math anxiety scale: applicability and usefulness: insights from the polish adaptation. Front. Psychol. 6:1833. doi: 10.3389/fpsyg.2015.01833, PMID: 26648893 PMC4663255

[ref15] Cipora K. Willmes K. Szwarc A. Nuerk H. C. (2017). Norms and validation of the online and paper-and-pencil versions of the abbreviated math anxiety scale (AMAS) for polish adolescents and adults. J. Numer. Cogn. 3, 667–693. doi: 10.5964/jnc.v3i3.121

[ref16] Cohen L. A. Limbers C. A. (2022). Factor structure and gender invariance of the abbreviated math anxiety scale (AMAS) in middle school students. Trends Psychol. 30, 788–807. doi: 10.1007/s43076-022-00167-6 PMC8937004 40477951

[ref17] Devine A. Fawcett K. Szucs D. Dowker A. (2012). Gender differences in mathematics anxiety and the relation to mathematics performance while controlling for test anxiety. Behav. Brain Funct. 8, 1–9. doi: 10.1186/1744-9081-8-33, PMID: 22769743 PMC3414752

[ref18] Dowker A. Bennett K. Smith L. (2012). Attitudes to mathematics in primary school children. Child Dev. Res. 2012, 1–8. doi: 10.1155/2012/124939

[ref19] Dowker A. Sarkar A. Looi C. Y. (2016). Mathematics anxiety: what have we learned in 60 years? Front. Psychol. 7:508. doi: 10.3389/fpsyg.2016.00508, PMID: 27199789 PMC4842756

[ref20] Faust M. W. (1996). Mathematics anxiety effects in simple and complex addition. Math. Cogn. 2, 25–62. doi: 10.1080/135467996387534

[ref21] Ferguson A. M. Maloney E. A. Fugelsang J. Risko E. F. (2015). On the relation between math and spatial ability: the case of math anxiety. Learn. Individ. Differ. 39, 1–12. doi: 10.1016/j.lindif.2015.02.007

[ref22] Field A. P. Evans D. Bloniewski T. Kovas Y. (2019). Predicting maths anxiety from mathematical achievement across the transition from primary to secondary education. R. Soc. Open Sci. 6:191459. doi: 10.1098/rsos.191459, PMID: 31827871 PMC6894589

[ref23] Fournier K. A. Couret J. Ramsay J. B. Caulkins J. L. (2017). Using collaborative two-stage examinations to address test anxiety in a large enrollment gateway course. Anat. Sci. Educ. 10, 409–422. doi: 10.1002/ase.1677, PMID: 28135034

[ref24] Ganley C. M. Vasilyeva M. (2014). The role of anxiety and working memory in gender differences in mathematics. J. Educ. Psychol. 106, 105–120. doi: 10.1037/a0034099

[ref25] Goetz T. Bieg M. Lüdke O. Pekrun R. Hall N. C. (2013). Do girls really experience more anxiety in mathematics? Psychol. Sci. 24, 2079–2087. doi: 10.1016/j.cedpsych.2012.09.001, PMID: 23985576

[ref26] Gunderson E. A. Park D. Maloney E. A. Beilock S. L. Levine S. C. (2018). Reciprocal relations among motivational frameworks, math anxiety, and math achievement in early elementary school. J. Cogn. Dev. 19, 21–46. doi: 10.1080/15248372.2017.1421538

[ref27] Haase V. G. Guimarães A. P. L. Wood G. (2019). “Math & emotion: the case of math anxiety” in International handbook of math difficulties: From the lab to the classroom. eds. Fritz A. Räsänen P. (São Paolo, Brazil: Springer)

[ref28] Harari R. R. Vukovic R. K. Bailey S. P. (2013). Mathematics anxiety in young children: an exploratory study. J. Exp. Educ. 81, 538–555. doi: 10.1080/00220973.2012.727888

[ref29] Hembree R. (1990). The nature, effects, and relief of anxiety mathematics. J. Res. Math. Educ. 21, 33–46. doi: 10.5951/jresematheduc.21.1.0033

[ref30] Hopko D. R. Mahadevan R. Bare R. L. Hunt M. K. (2003). The abbreviated math anxiety scale (AMAS) construction, validity, and reliability. Assessment 10, 178–182. doi: 10.1177/1073191103010002008, PMID: 12801189

[ref31] Hu L. T. Bentler P. M. (1999). Cutoff criteria for fit indexes in covariance structure analysis: conventional criteria versus new alternatives. Struct. Equ. Model. Multidiscip. J. 6, 1–55. doi: 10.1080/10705519909540118

[ref32] Ismatullina V. Adamovich T. Zakharov I. Vasin G. Voronin I. (2022). The place of gender stereotypes in the network of cognitive abilities, self-perceived ability and intrinsic value of School in School Children Depending on sex and preferences in STEM. Behav. Sci. 12:75. doi: 10.3390/bs12030075, PMID: 35323394 PMC8944967

[ref33] Johnston-Wilder S. Brindley J. Dent P. (2014). Technical report: A survey of mathematics anxiety and mathematical resilience amongst existing apprentices (London: Gatsby Charitable Foundation).

[ref34] Kiessler R. C. Berglund P. Demler O. Jin R. Merikangas K. Walters E. (2005). Lifetime prevalence and age-of-onset distributions of DSM-IV disorders in the national comorbidity survey replication. Arch. Gen. Psychiatry 62, 593–602. doi: 10.1001/archpsyc.62.6.593, PMID: 15939837

[ref35] Kline R. B. (2005). Principles and practice of structural equation modeling, (New York: The Guilford Press).

[ref36] Lu Y. Li Q. Patrick H. Mantzicopoulos P. (2021). “Math gives me a tummy ache!” mathematics anxiety in kindergarten. J. Exp. Educ. 89, 362–378. doi: 10.1080/00220973.2019.1680518

[ref37] Luo W. Hogan D. Tan L. S. Kaur B. Ng P. T. Chan M. (2014). Self-construal and students’ math self-concept, anxiety and achievement: an examination of achievement goals as mediators. Asian J. Soc. Psychol. 17, 184–195. doi: 10.1111/ajsp.12058

[ref38] Ma X. (1999). A meta-analysis of the relationship between anxiety toward mathematics and achievement in mathematics. J. Res. Math. Educ. 30, 520–540. doi: 10.2307/749772

[ref39] Ma X. Xu J. (2004). The causal ordering of mathematics anxiety and mathematics achievement: a longitudinal panel analysis. J. Adolesc. 27, 165–179. doi: 10.1016/j.adolescence.2003.11.003, PMID: 15023516

[ref40] Malanchini M. Rimfeld K. Shakeshaft N. G. Rodic M. Schofield K. Selzam S.. (2017). The genetic and environmental aetiology of spatial, mathematics and general anxiety. Sci. Rep. 7:42218. doi: 10.1038/srep42218, PMID: 28220830 PMC5318949

[ref41] Mallow J. V. (2006). “Science anxiety: research and action” in Handbook of college science teaching. eds. Mintzes J. J. Leonard W. H. (Arlington, Virginia, USA: NSTApress), 3–14.

[ref42] Maloney E. A. Ansari D. Fugelsang J. A. (2011). Rapid communication: the effect of mathematics anxiety on the processing of numerical magnitude. Q. J. Exp. Psychol. 64, 10–16. doi: 10.1080/17470218.2010.533278 21113858

[ref43] Maloney E. A. Risko E. F. Ansari D. Fugelsang J. (2010). Mathematics anxiety affects counting but not subitizing during visual enumeration. Cognition 114, 293–297. doi: 10.1016/j.cognition.2009.09.013 19896124

[ref44] Maloney E. A. Waechter S. Risko E. F. Fugelsang J. A. (2012). Reducing the sex difference in math anxiety: the role of spatial processing ability. Learn. Individ. Differ. 22, 380–384. doi: 10.1016/j.lindif.2012.01.001

[ref45] Martín-Puga M. E. Justicia-Galiano M. J. Gómez-Pérez M. M. Pelegrina S. (2022). Psychometric properties, factor structure, and gender and educational level invariance of the abbreviated math anxiety scale (AMAS) in Spanish children and adolescents. Assessment 29, 425–440. doi: 10.1177/1073191120980064, PMID: 33334166

[ref46] McLean C. P. Anderson E. R. (2009). Brave men and timid women? A review of the gender differences in fear and anxiety. Clin. Psychol. Rev. 29, 496–505. doi: 10.1016/j.cpr.2009.05.003, PMID: 19541399

[ref47] Meece J. L. Wigfield A. Eccles J. S. (1990). Predictors of math anxiety and its influence on young adolescents' course enrollment intentions and performance in mathematics. J. Educ. Psychol. 82, 60–70. doi: 10.1037/0022-0663.82.1.60

[ref48] Megreya A. M. Szűcs D. Moustafa A. A. (2021). The abbreviated science anxiety scale: psychometric properties, gender differences and associations with test anxiety, general anxiety and science achievement. PLoS One 16:e0245200. doi: 10.1371/journal.pone.0245200, PMID: 33577578 PMC7880483

[ref49] Miller H. Bichsel J. (2004). Anxiety, working memory, gender, and math performance. Pers. Individ. Differ. 37, 591–606. doi: 10.1016/j.paid.2003.09.029

[ref50] Milovanović I. Branovački B. (2021). Adaptation and psychometric evaluation of modified abbreviated math anxiety scale for children in Serbia. Int. J. Sci. Math. Educ. 19, 579–598. doi: 10.1007/s10763-020-10066-w

[ref51] Núñez-Peña M. I. Guilera G. Suárez-Pellicioni M. (2014). The single-item math anxiety scale: an alternative way of measuring mathematical anxiety. J. Psychoeduc. Assess. 32, 306–317. doi: 10.1177/0734282913508528

[ref52] Nunnally J. C. (1978). Psychometric theory, (New York, USA: McGraw).

[ref53] Orbach L. Herzog M. Fritz A. (2020). State-and trait-math anxiety and their relation to math performance in children: the role of core executive functions. Cognition 200:104271. doi: 10.1016/j.cognition.2020.104271, PMID: 32428703

[ref54] Park D. Ramirez G. Beilock S. L. (2014). The role of expressive writing in math anxiety. J. Exp. Psychol. Appl. 20, 103–111. doi: 10.1037/xap0000013, PMID: 24708352

[ref55] Pekrun R. (2006). The control-value theory of achievement emotions: assumptions, corollaries, and implications for educational research and practice. Educ. Psychol. Rev. 18, 315–341. doi: 10.1007/s10648-006-9029-9

[ref56] Plake B. S. Parker C. S. (1982). The development and validation of a revised version of the mathematics anxiety rating scale. Educ. Psychol. Meas. 42, 551–557. doi: 10.1177/001316448204200218

[ref57] Primi C. Donati M. A. Izzo V. A. Guardabassi V. O’Connor P. A. Tomasetto C.. (2020). The Early Elementary School Abbreviated Math Anxiety Scale (the EES-AMAS): A New Adapted Version of the AMAS to Measure Math Anxiety in Young Children. Front. Psychol. 11:1014. doi: 10.3389/fpsyg.2020.01014, PMID: 32528380 PMC7253683

[ref58] Richardson F. C. Suinn R. M. (1972). The mathematics anxiety rating scale: psychometric data. J. Couns. Psychol. 19, 551–554. doi: 10.1037/h0033456

[ref59] Sadiković S. Milovanović I. Oljača M. (2018). Another psychometric proof of the abbreviated math anxiety scale usefulness: IRT analysis. Primenj. Psihol. 11, 301–323. doi: 10.19090/pp.2018.3.301-323

[ref60] Schillinger F. L. Vogel S. E. Diedrich J. Grabner R. H. (2018). Math anxiety, intelligence, and performance in mathematics: insights from the German adaptation of the abbreviated math anxiety scale (AMAS-G). Learn. Individ. Differ. 61, 109–119. doi: 10.1016/j.lindif.2017.11.014

[ref61] Schmader T. Johns M. Forbes C. (2008). An integrated process model of stereotype threat effects on performance. Psychol. Rev. 115, 336–356. doi: 10.1037/0033-295X.115.2.336, PMID: 18426293 PMC2570773

[ref62] Spelke E. (2005). Sex differences in intrinsic aptitude for mathematics and science? a critical review. Am. Psychol. 60, 950–958. doi: 10.1037/0003-066X.60.9.950, PMID: 16366817

[ref63] Spielberger C. D. Gorsuch R. L. Lushene R. (1970). Manual for the state-trait anxiety inventory self-evaluation questionnaire (California: Consulting Psychologists Press).

[ref64] Szczygiel M. (2020). Gender, general anxiety, math anxiety and math achievement in early school-age children. Issues Educ. 30, 1126–1142. doi: 10.3316/informit.465488906598804

[ref65] Tomasetto C. (2019). “Gender stereotypes, anxiety, and math outcomes in adults and children” in Mathematics anxiety. eds. Mammarella I. C. Caviola S. Dowker A. (London, NY: Routledge), 178–189.

[ref66] Wang Z. Hart S. A. Kovas Y. Lukovski S. Soden B. Thompson L. A.. (2014). Who is afraid of math? Two sources of genetic variance for mathematical anxiety. J. Child Psychol. Psychiatry 55, 1056–1064. doi: 10.1111/jcpp.12224, PMID: 24611799 PMC4636726

[ref67] Wu S. S. Barth M. Amin H. Malcarne V. Menon V. (2012). Math anxiety in second and third graders and its relation to mathematics achievement. Front. Psychol. 3:162. doi: 10.3389/fpsyg.2012.00162, PMID: 22701105 PMC3369194

[ref68] Xie F. Xin Z. Chen X. Zhang L. (2019). Gender difference of Chinese high school students’ math anxiety: the effects of self-esteem, test anxiety and general anxiety. Sex Roles 81, 235–244. doi: 10.1007/s11199-018-0982-9

[ref69] Young C. B. Wu S. S. Menon V. (2012). The neurodevelopmental basis of math anxiety. Psychol. Sci. 23, 492–501. doi: 10.1177/0956797611429134, PMID: 22434239 PMC3462591

[ref70] Zhang J. Zhao N. Kong Q. P. (2019). The relationship between math anxiety and math performance: a meta-analytic investigation. Front. Psychol. 10:1613. doi: 10.3389/fpsyg.2019.01613, PMID: 31447719 PMC6692457

